# Central rib and the nutritive value of leaves in forage grasses

**DOI:** 10.1038/s41598-021-84844-z

**Published:** 2021-03-08

**Authors:** Larissa Fernanda Garcia, Guilherme Portes Silva, Eliana Vera Geremia, Laura Beatriz Lima Goulart, Carlos Tadeu dos Santos Dias, Sila Carneiro da Silva

**Affiliations:** 1 Animal Science Department, University of Sao Paulo, “Luiz de Queiroz” College of Agriculture (USP/ESALQ), Av. Pádua Dias, 11, Piracicaba, Sao Paulo 13418-900 Brazil; 2 Math, Chemistry and Statistics Department, University of Sao Paulo, “Luiz de Queiroz” College of Agriculture (USP/ESALQ), Av. Pádua Dias, 11, Piracicaba, Sao Paulo 13418-900 Brazil; 3 Soil Science Department, University of Ceara, Campus do Pici. Bloco 807, Av. Mister Hull, s/n, Fortaleza, CE 60455-760 Brazil

**Keywords:** Ecophysiology, Plant development, Leaf development

## Abstract

In grasses, leaf expansion and central rib growth occur in a non-proportional manner, with potential implications to the nutritive value of leaves. The objective of this study was to evaluate the relationship among blade length, percentage of central rib, anatomical characteristics and the nutritive value along the length of leaf blades of different sizes and hierarchical order of insertion on the tiller axis of Napier elephant grass (*Pennisetum purpureum* Schum. cv. Napier). Two experiments were carried out with isolated growing plants during the summer of 2017 (January to March). Central rib mass increased linearly with the increase in leaf blade mass and its percentage relative to blade mass decreased from the base to the tip of the leaf. There were no significant variations in anatomical characteristics along the length of leaf blades when central rib was not taken into account. The central rib showed negative relationship with nutritive value. The apical portions of long leaves showed similar digestibility to short leaves. The multivariate analysis of Cluster and Principal Components grouped the response variables according to leaf hierarchical order, final blade length and percentage of structural tissues, highlighting the relationship between leaf size, structural tissues and nutritive value.

## Introduction

In forage grasses, leaves are usually the morphological component with highest nutritive and feeding value. Their development start as primordia located on opposite sides of tiller apical meristems^1^, with blade length varying according to their order of appearance on the tiller axis (hierarchic order of insertion) and greatest values normally recorded on leaves of intermediate levels of insertion^2, 3^. Morphologically, the leaf blade is normally linear or lanceolate shaped, with characteristic parallel ribs^4^, the main one being the central rib, which provides additional structural support to the leaf^5^. Leaf growth is developmentally coordinated with the growth of the major veins, given that the bulk of leaf expansion results from leaf length. For that reason, having a structurally reinforced central rib (i.e. more diameter growth of the midvein with greater elongation of the blade) is biomechanically necessary and reduces the increasing pressure required to conduct water with increasing path length^6^.

Anatomically, the leaf blade is comprised of vascular tissue (vascular bundles), represented by xylem and phloem cells; ground or support tissue, represented by parenchyma, collenchyma and sclerenchyma which in grasses is frequently associated with vascular tissue; and assimilatory tissue, represented by mesophyll cells^7^. These tissues are covered on both sides of the blade (abaxial and adaxial) by the epidermis that, in turn, is covered externally by the cuticle. At the same stage of development, leaves show an anatomical tissue gradient depending on their level of insertion on the tiller according to which blades and sheaths from leaves ranked higher on the tiller axis (closer to the apical meristem) have greater proportion of sclerenchyma and vascular tissue relative to those ranked lower (closer to the base of the tiller)^8^.

Leaf blades of higher insertion levels are usually longer providing that there is no elongation of the the apical meristem (internode elongation)^9–11^. As a result, they may have greater proportion of sclerenchyma and vascular tissue due to the need for structural support to maintain its erect architecture for intercepting the incident light^12^. The sclerenchyma along with the xylem cells are important to provide support to the erect growth of the leaves, particularly in warm and dry environments^13, 14^. These tissues are formed by cells with thickened secondary walls and are the main determinants of reduced herbage quality^15, 16^. In this context, as the central rib usually contains large proportions of vascular tissue and sclerenchyma, it has digestibility considerably smaller than the blade^17^. However, both shape and distribution of central rib mass vary along the leaf length, since central rib as well as second order parallel veins taper from the base to the tip during leaf growth, resulting in leaf tips narrower than leaf base; the lanceolate shape^4, 6, 18^.

Although highly significant correlations among nutritive value, morphological and anatomical characteristics have been reported in the literature^19–21^, studies evaluating the sole influence of the central rib on those relationships are almost no existent. In this context, given the relationship between herbage nutritive value and the anatomic composition of plant tissues^20^, the study regarding leaf anatomy and morphology may provide an useful tool for discriminating promising genotypes in qualitative terms in the early phases of evaluation of new forage materials^22^. It may also help with the identification of factors limiting intake^23^ and the results used to develop plant three-dimensional growth models that could be used to predict nutritive value and nutrient intake by grazing animals as well as to define targets for grazing management strategies.

Against that background, it was hypothesised that in isolated forage plants under free growth conditions leaf expansion and central rib growth occur in a non-proportional manner, affecting the morphology and the anatomy of the leaf blade and interfering with nutritive value. Defoliations removing only the apical portion of the leaves (the last third close to the tip), a condition where the central rib represents a small proportion of the blade mass, result in the removal of herbage with similar nutritive value of small, young leaves, despite the difference in chronological and physiological age between them.

In this study Napier elephant grass *(Pennisetum purpureum* Schum. cv. Napier), an important tropical forage grass species for pastoral systems and bioenergy production, was used as model plant because of its large leaf size and easy of vegetative propagation. The objective was to evaluate the relationship among final blade length, percentage of central rib mass and the anatomical and chemical (nutritive value) characteristics along leaves of different sizes and hierarchical order of insertion on the axis (phytomers 8 to 16) of basal tillers of this important forage grass.

## Results

### Leaf morphology

Leaf blade length and the percentage of central rib mass increased as the level of leaf insertion on the tiller increased (closer to the apical meristem). On lower hierarchical order leaves, smaller leaves, 70% of the total mass corresponded to blade and 30% to central rib. On higher hierarchical order leaves, bigger leaves, the corresponding values were 60 and 40%, respectively (Table 1).

**Table 1:** Final blade length and blade and central rib mass of Napier elephant grass leaves.

Phytomer	BL	SEM	CRM	BM	SEM*	CRM	BM
	(cm)		(mg)	(mg)		(% total WBM)**	(% total WBM )**
8	28.4	1.16	43	100	5.9	30	70
9	34.2	2.13	63	145	83.8	30	70
10	41.8	0.79	107	192	13.9	36	64
11	46.1	3.30	135	264	33.8	34	66
12	55.3	3.13	189	318	22.9	37	63
13	60.6	1.25	238	380	13.1	39	61
14	61.1	3.25	234	383	30.0	38	62
15	66.8	3.14	299	500	46.6	37	63
16	73.9	0.70	368	525	15.3	41	59

*BL* blade length; *SEM* standard error of the mean; *CRM* central rib mass; *BM* blade mass; *Standard error of the mean for CRM and BM.

**Percentage of the whole leaf blade mass (WBM).

**Figure 1:**
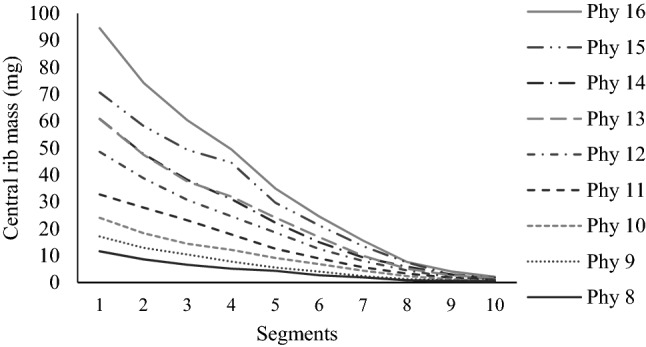
Central rib mass along the blade of leaves with contrasting levels of insertion on the tiller axis (phytomers) of Napier elephant grass.

**Figure 2:**
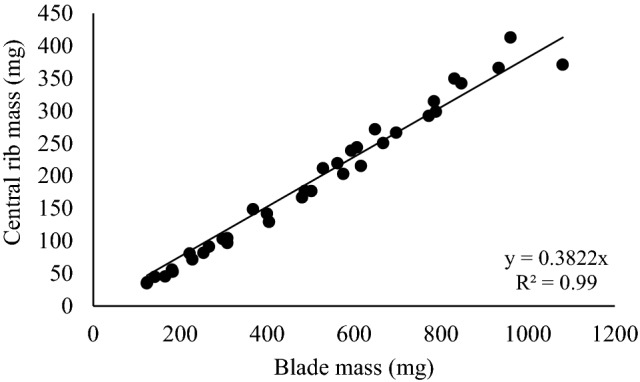
Relationship between whole leaf central rib mass (mg) and blade mass (mg) of Napier elephant grass leaves.

The central rib was present along the entire length of the blades, but corresponded to greater percentage of the blade mass closer to the leaf base (segments 1 and 2) and minimal percentages closer to the tip (segments 9 and 10) (Fig. 1). The proportion of central rib in the whole blade mass increased in longer leaves (phytomers 14, 15 and 16), with values varying from 11.6 to 94.5 mg for the basal segment (segment 1) and 0.5 to 2.2 mg for the apical segment (segment 10) on leaves 8 and 16, respectively. When added the values of central rib and blade mass from all ten segments (values for the whole leaves from phytomers 8 to 16), it was observed that central rib growth increased linearly with the increase in blade mass (y = 0.3822x, Pr >|t|= < 0.0001, R^2^ = 0.99) (Fig. 2).

### Leaf anatomy

There was a slight variation in the percentage of anatomical tissues on the leaf blades as their level of insertion on the tiller axis increased. The percentage of both abaxial and adaxial epidermis slightly decreased and that of vascular tissue and sclerenchyma slightly increased as leaf size increased from phytomers 8 to 16 (Table 2). On the other hand, anatomical characteristics were quite stable along the blades (from the basal to the tip portion) for all the studied phytomers (Table 3).

**Table 2:** Linear regression between final blade length and the percentage of anatomical tissues along the elephant grass leaves (phytomers 8 to 16).

Component (% total area)	Blade length						
	α	SE (α)	Pr >|t| (α)	β	SE (β)	Pr >|t| (β)	R^2^
EPIada	23.205	0.924	< .0001	− 0.085	0.018	< .0001	0.118
EPIaba	17.637	0.554	< .0001	− 0.079	0.011	< .0001	0.282
MES	42.356	1.173	< .0001	0.051	0.022	0.0221	0.031
PSVB	12.752	0.368	< .0001	0.013	0.007	0.0614	0.025
VT	4.328	0.433	< .0001	0.078	0.008	< .0001	0.354
SCL	0.112	0.161	0.4863	0.014	0.003	< .0001	0.117

*MES* mesophyll, *PSVB* parenchymatic sheath of vascular bundles, *VT* vascular tissue, *SCL* sclerenchyma, *EPIada* adaxial epidermis and *EPIaba* abaxial epidermis. *α* intercept, *SE* standard error, *β* slope.

**Table 3:** Linear regression between segments of the leaf blade (basal, mid-basal, middle, mid-apical and apical portions) and the percentage of anatomical tissues of leaves of contrasting insertion levels along the tiller axis of elephant grass.

Phytomer	α	SE (α)	Pr >|t| (α)	β	SE (β)	Pr >|t| (β)	R^2^
	**Mesophyll**						
8	48.131	10.072	0.0002	− 0.205	0.353	0.5692	0.018
9	39.364	12.199	0.0053	0.176	0.376	0.6469	0.013
10	49.626	7.777	< .0001	− 0.105	0.192	0.5890	0.017
11	56.029	8.814	< .0001	− 0.275	0.188	0.1629	0.118
12	46.170	8.406	< .0001	0.018	0.157	0.9107	0.001
13	45.444	9.071	< .0001	− 0.002	0.154	0.9897	0.000
14	13.412	52.482	0.8014	51.776	0.810	0.5310	0.024
15	41.671	9.132	0.0003	0.026	0.140	0.8551	0.002
16	30.029	10.872	0.0133	0.234	0.159	0.1590	0.113
	**Parenchymatic sheath of vascular bundles**						
8	16.179	4.029	0.0008	− 0.098	0.141	0.4947	0.026
9	13.069	2.555	0.0001	− 0.012	0.079	0.8773	0.002
10	10.987	2.288	0.0001	0.053	0.056	0.3557	0.048
11	13.381	4.227	0.0060	0.003	0.090	0.9720	0.000
12	10.388	3.596	0.0107	0.049	0.067	0.4763	0.032
13	13.374	2.786	0.0002	− 0.001	0.047	0.9849	0.000
14	9.271	15.279	0.5544	0.064	0.236	0.7888	0.006
15	12.905	3.021	0.0006	0.023	0.046	0.6308	0.015
16	9.266	2.930	0.00570	0.060	0.043	0.1818	0.102
	**Vascular tissue**						
8	2.519	2.912	0.3985	0.128	0.102	0.2264	0.080
9	1.494	3.893	0.7061	0.176	0.120	0.1623	0.118
10	3.944	3.139	0.225	0.083	0.077	0.2960	0.061
11	6.463	3.089	0.0527	0.038	0.066	0.5701	0.021
12	3.158	2.872	0.2877	0.095	0.054	0.0952	0.164
13	7.213	2.125	0.0034	0.035	0.036	0.3499	0.052
14	18.236	15.348	0.2511	− 0.135	0.237	0.5762	0.019
15	10.036	4.396	0.0364	0.008	0.067	0.9101	0.001
16	2.425	6.089	0.6954	0.090	0.089	0.3251	0.057
	**Sclerenchyma**						
8	− 0.884	0.994	0.3856	0.046	0.035	0.1995	0.090
9	− 0.152	1.246	0.9044	0.023	0.038	0.5541	0.022
10	− 0.005	1.034	0.9962	0.014	0.026	0.5914	0.016
11	0.800	1.543	0.6112	0.000	0.033	0.9988	0.000
12	− 0.407	1.604	0.8031	0.024	0.030	0.4300	0.039
13	1.998	1.017	0.066	− 0.016	0.017	0.3593	0.050
14	7.968	7.083	0.2762	− 0.107	0.109	0.3401	0.054
15	5.047	1.295	0.0013	− 0.057	0.020	0.0107	0.343
16	0.598	1.602	0.7134	0.005	0.023	0.8366	0.003

	**Adaxial epidermis**						
8	21.469	8.030	0.0155	− 0.004	0.281	0.9878	0.000
9	29.224	10.330	0.0121	− 0.274	0.318	0.4025	0.044
10	24.393	7.236	0.0034	− 0.128	0.178	0.4822	0.028
11	12.477	7.928	0.1351	0.163	0.169	0.3494	0.055
12	25.269	5.929	0.0006	− 0.151	0.111	0.1922	0.104
13	19.293	6.934	0.0128	− 0.015	0.118	0.8981	0.001
14	39.117	33.362	0.2572	− 0.344	0.515	0.5131	0.026
15	15.950	7.546	0.0506	0.024	0.116	0.8382	0.003
16	34.161	9.636	0.0032	− 0.232	0.139	0.1182	0.165
	**Abaxial epidermis**						
8	12.592	5.345	0.03	0.133	0.187	0.4865	0.027
9	17.018	6.620	0.0205	− 0.089	0.204	0.6687	0.012
10	11.063	3.703	0.0079	0.083	0.091	0.3778	0.044
11	12.164	5.550	0.0472	0.038	0.120	0.7541	0.008
12	17.973	2.193	< .0001	− 0.082	0.041	0.0685	0.250
13	12.691	2.722	0.0002	0.000	0.046	0.9928	0.000
14	2.321	26.545	0.9314	0.158	0.409	0.7047	0.009
15	14.382	3.855	0.0018	− 0.023	0.059	0.7031	0.009
16	16.506	6.141	0.0177	− 0.069	0.089	0.4480	0.042

*α* intercept, *SE* standard error, *β* slope.

**Figure 3:**
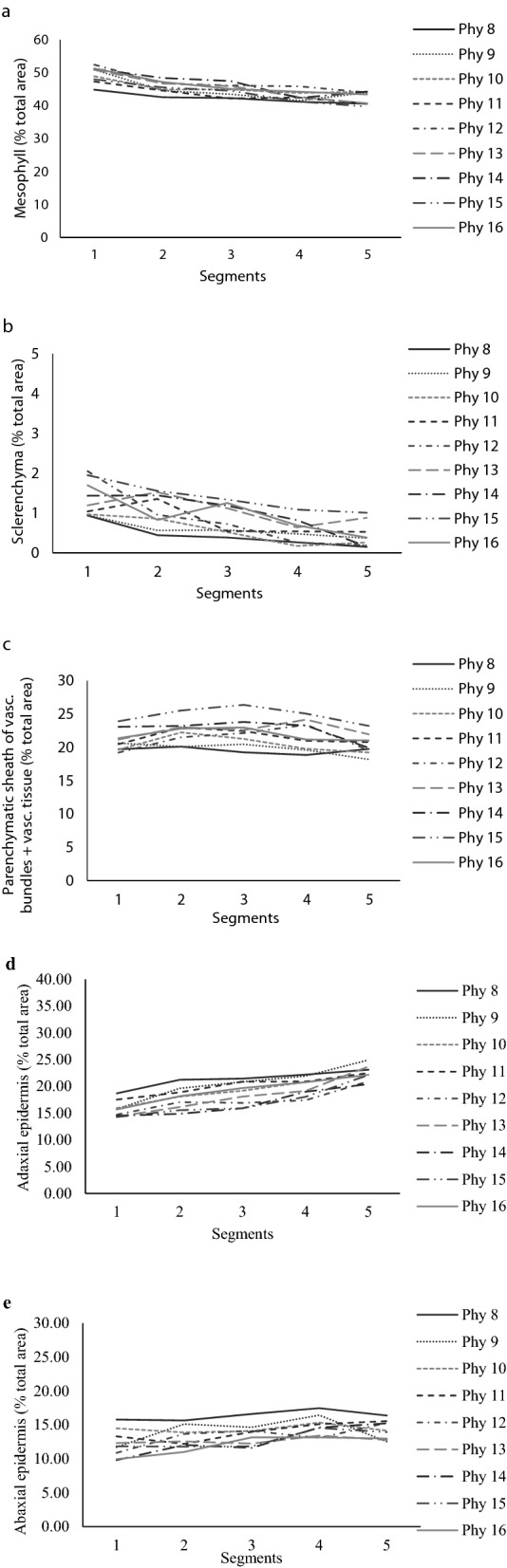
Percentage of anatomical tissues relative to blade total cross-section area of leaves with contrasting levels of insertion on the tiller axis (phytomers) of Napier elephant grass. (**a**–**e**): mesophyll, sclerenchyma, parenchymatic sheath of vascular bundles + vascular tissue, adaxial and abaxial epidermis, respectively. Phytomers were individually harvested soon after leaf complete expansion.

**Figure 4:**
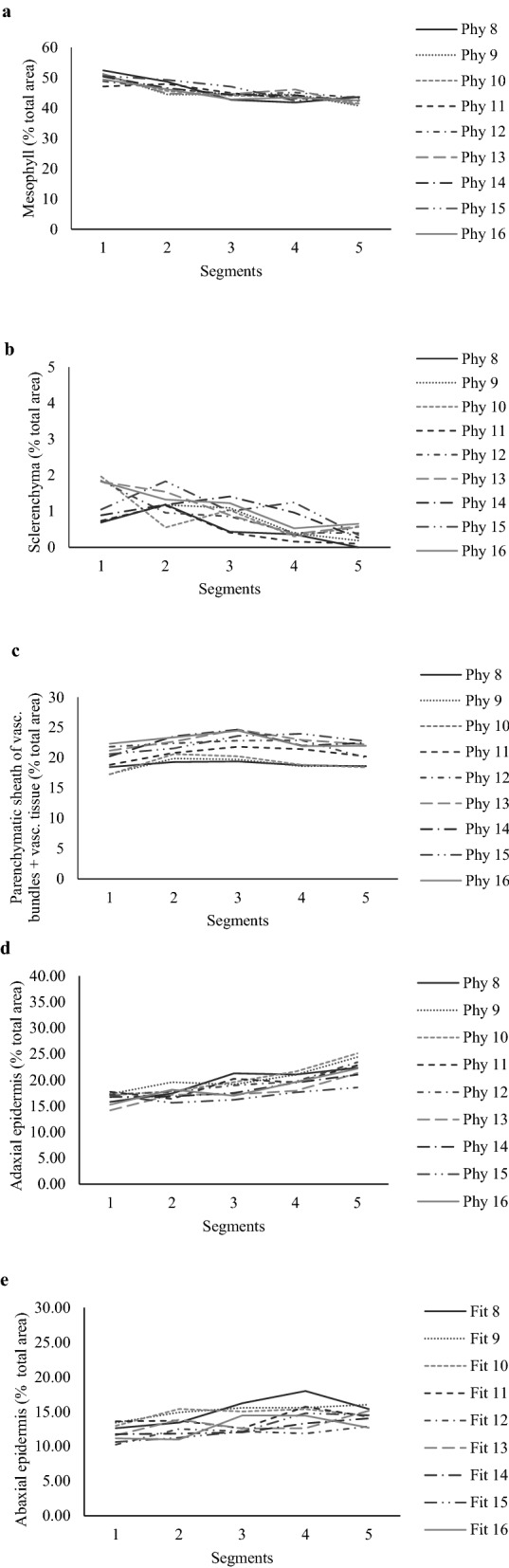
Percentage of anatomical tissues relative to blade total cross-section area of leaves with contrasting levels of insertion on the tiller axis (phytomers) of Napier elephant grass. (**a**–**e**): mesophyll, sclerenchyma, parenchymatic sheath of vascular bundles + vascular tissue, adaxial and abaxial epidermis, respectively. Phytomers were all harvested after complete expansion of leaf 16.

Only a slight variation in anatomical characteristics was observed along the length of the leaves as described above for leaf morphology. For the percentage of mesophyll, a common pattern of variation was detected for all phytomers, according to which greater and smaller percentages were recorded on the basal and mid-apical/apical portions of the leaves, with variations of 45–52; 43–48; 42–47; 41–46 and 40–44% of the total area of the segments 1, 2, 3, 4 and 5, respectively (Fig. 3a). The same pattern was observed for sclerenchyma percentage, with variations of 0.9–2.0; 0.4–1.5; 0.3–1.3; 0.2–1.0 and 0.1–1.0% of the total area of the segments 1, 2, 3, 4 and 5, respectively (Fig. 3b). For the parenchymatic sheath of the vascular bundles and vascular tissue, greater percentage was observed on the mid-basal and middle segments (2 and 3), with variations from 18 to 26% of the total area of the segments (Fig. 3c). For the adaxial epidermis, greater percentage was observed on the apical portion (segment 5), values ranging from 20 to 25% of the total area of the segments (Fig. 3d). For the abaxial epidermis greater percentage was observed on the mid-apical/apical portions of the leaves, values ranging from 12 to 17% of the total area of the segments (Fig. 3e). The response patterns of anatomical composition relative to plant age (harvest of leaves when the 16th phytomer completed expansion and exposed the ligule) were similar to those described for individual phytomers soon after their complete expansion. The variations were: mesophyll—47–52; 44–49; 43–47; 42–46 and 41–44% of the total area of the segments (Fig. 4a); sclerenchyma—0.7–2.0; 0.5–1.8; 0.4–1.4; 0.2–1.2 and 0–0.6% of the total area of the segments (Fig. 4b), both for segments 1, 2, 3, 4 and 5, respectively; parenchymatic sheath of the vascular bundles and vascular tissue—17–25% of the total area of segments; adaxial epidermis—19–25% and abaxial epidermis–12–18% of the total area of segments (only 1% difference from the corresponding variations for individual phytomers soon after their complete expansion) (Fig. 4c,d,e).

Crude protein (CP) content and in vitro dry matter digestibility (IVDMD) varied along the leaf blades (*P* < 0.0001). The apical portion (segment 5) of phytomer 16, which presented the greatest lengths and smallest percentages of central rib, showed greatest IVDMD (85.8%), since it was the youngest leaf on the tiller, with wider blade and more digestible tissues (i.e. epidermis and spongy tissues). Similar IVDMD values were recorded for segments 1, 2, 3 and 4 of phytomers 8, 9 and 10; segments 2, 3 and 4 of phytomer 11; segments 3, 4 and 5 of phytomers 12, 13, 14 and 15 and segment 4 of phytomer 16 (Table 4). Smaller IVDMD values were recorded on the apical portion of small, old leaves (e.g. low hierarchical order on the tiller axis). In relation to CP percentage, greatest value was recorded on the apical segment (segment 5) of phytomer 16 (Table 5), which did not differ from the mid-apical segment (segment 4) of phytomer 10, segments 4 and 5 of phytomers 11, 12, 13, 14 and 15 and segments 3 and 4 of phytomer 16. Overall, greater CP percentages were observed on the apical portions of the leaf blades of phytomers with high levels of insertion on the tiller axis (closer to the apical meristem) and smaller percentages on the basal portions of all phytomers.

**Table 4:** In vitro dry matter digestibility (IVDMD) along the blades of Napier elephant grass leaves.

Phytomer	Segments				
	1	2	3	4	5
8	*83.5 abcd*	*83.9 abc*	*83.6 abcd*	*82.2 abcdef*	77.9 gh
	*(0.93)*	*(0.47)*	*(0.98)*	*(0.81)*	(0.57)
9	*83.1 abcd*	*83.8 abcd*	*85.0 ab*	*83.2 abcd*	80.1 cdefgh
	*(0.20)*	*(0.36)*	*(1.50)*	*(0.34)*	(0,77)
10	*82.0 abcdefg*	*83.2 abcd*	*83.2 abcd*	*81.7 abcdefg*	80.3 cdefg
	*(0.09)*	*(0.47)*	*(0.47)*	*(0.63)*	(0.77)
11	81.2 bcdefg	*83.5 abcd*	*82.5 abcdef*	*83.5 abcd*	79.9 cdefgh
	(0.82)	*(0.28)*	*(0.41)*	*(1.53)*	(1.15)
12	81.0 bcdefg	81.5 bcdefg	*82.8 abcde*	*82.6 abcdef*	*82.6 abcdef*
	(0.83)	(0.57)	*(0.41)*	*(1.84)*	*(1.17)*
13	78.5 fgh	81.1 bcdefg	*82.6 abcdef*	*82.2 abcdefg*	*81.9 abcdefg*
	(0.41)	(0.99)	*(0.81)*	*(1.27)*	*(0.19)*
14	79.6 defgh	79.6 defgh	*81.8 abcdefg*	*82.7 abcdef*	*83.4 abcd*
	(0.35)	(0.65)	*(0.62)*	*(0.49)*	*(0.81)*
15	75.9 h	78.8 efgh	*81.7 abcdefg*	*83.7 abcd*	*82.6 abcdef*
	(1.29)	(0.27)	*(0.74)*	*(1.21)*	*(0.80)*
16	78.7 efgh	78.5 fgh	80.9 bcdefg	*81.7 abcdefg*	*85.8 a*
	(0.95)	(0.64)	(0.77)	*(0.71)*	*(1.32)*

Means followed by the same letters are not different (*P* > 0.05). Values in parentheses correspond to the standard error of the mean. The highlights in italics indicate similar means.

**Table 5:** Crude protein (CP) percentage along the blade of Napier elephant grass leaves.

Phytomer	Segments				
	1	2	3	4	5
8	11.6o	14 klmn	15.1 ghijk	15.8 fghijk	15.5 fghijk
	(0.40)	(0.39)	(0.42)	(0.32)	(0.86)
9	12.0 no	14.8 ijklm	16.4 defghij	16.8 cdefghi	16.4 defghij
	(0.32)	(0.25)	(0.46)	(0.27)	(0.14)
10	12.6 mno	15.3 fghijk	16.0 efghijk	*18.1 abcde*	17.3 bcdefg
	(0.38)	(0.62)	(0.07)	*(0.18)*	(0.18)
11	12.7 lmno	14.9 hijkl	17.3 bcdefg	*18.5 abcd*	*17.6 abcdef*
	(0.52)	(0.11)	(0.75)	*(0.68)*	*(0.29)*
12	11.8 no	14.5 jklm	17.4 bcdef	*18.3 abcde*	*18.4 abcd*
	(0.90)	(0.69)	(0.85)	*(0.66)*	*(0.90)*
13	11.3 o	14 klmn	16.5 defghij	*19.1 abc*	*18.6 abcd*
	(0.48)	(0.16)	(0.37)	*(0.42)*	*(0.67)*
14	11.5 o	14.6 ijklm	17.3 bcdefg	*18.6 abcd*	*18.5 abcd*
	(0.56)	(0.55)	(0.65)	*(0.28)*	*(0.44)*
15	11.8 no	14.9 hijkl	17.2 bcdefgh	*19.2 ab*	*19.5 ab*
	(0.30)	(0.54)	(0.27)	*(0.27)*	*(0.57)*
16	11.0 o	14.4 jklm	*17.6 abcdef*	*19.2 ab*	*19.9 a*
	(0.16)	(0.02)	*(0.42)*	*(0.31)*	*(0.71)*

Means followed by the same letters are not different (*P* > 0.05). Values in parentheses correspond to the standard error of the mean. The highlights in italics indicate similar means.

### Principal components analysis (PCA)

**Figure 5:**
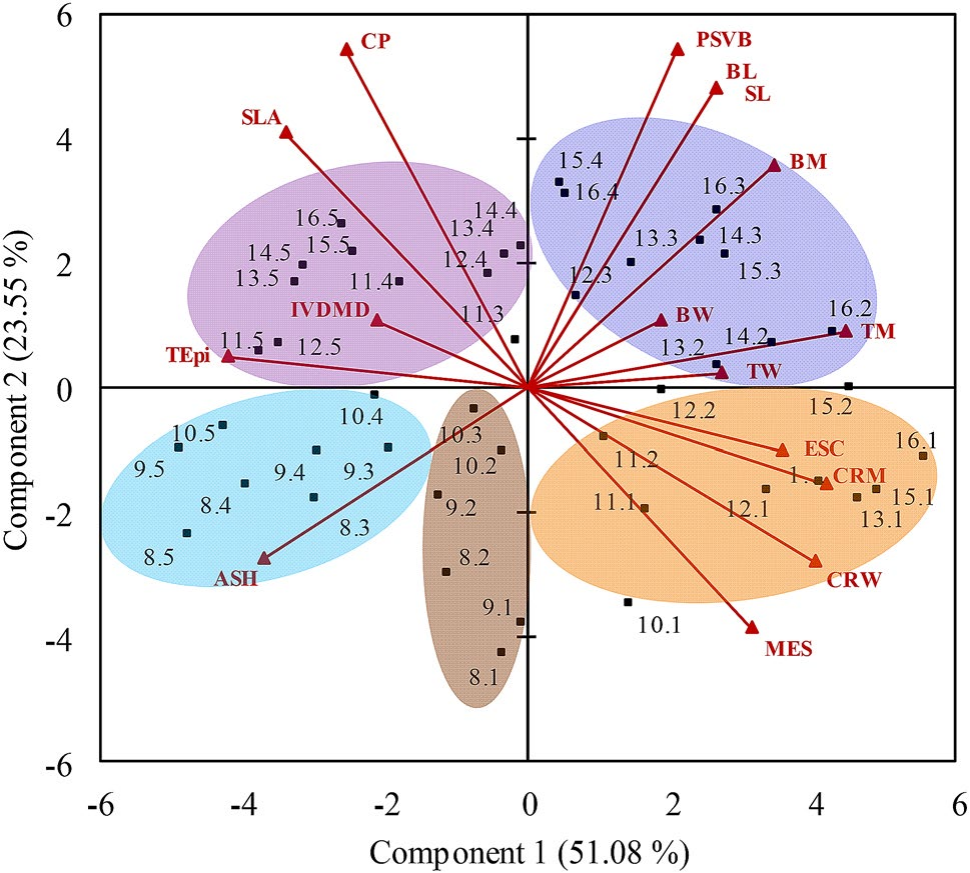
Biplot PC1 × PC2 for morphological (blade length (BL), segment length (SL), total width (TW), blade width (BW), central rib width (CRW), total mass (TM), blade mass (BM), central rib mass (CRM) and specific leaf area (SLA)), anatomical (sclerenchyma (SCL), parenchymatic sheath of vascular bundles + vascular tissue (PSVB), mesophyll (MES) and total epidermis (TEpi)) and nutritive value (crude protein (CP), in vitro dry matter digestibility (IVDMD) and ash (ASH)) characteristics of leaves with contrasting levels of insertion on the tiller axis (phytomers) of Napier elephant grass. Points represent the phytomers with their respective segments and arrows represent the vector for each variable.

**Figure 6:**
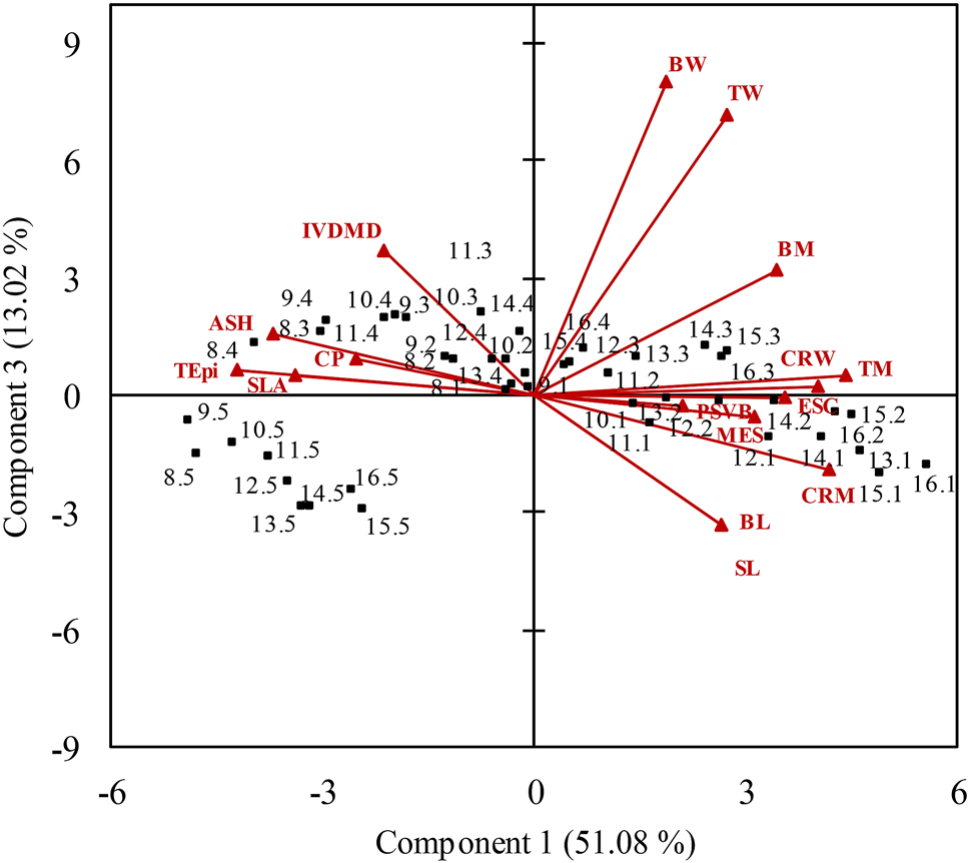
Biplot PC1 × PC3 for morphological (blade length (BL), segment length (SL), total width (TW), blade width (BW), central rib width (CRW), total mass (TM), blade mass (BM), central rib mass (CRM) and specific leaf area (SLA)), anatomical (sclerenchyma (SCL), parenchymatic sheath of vascular bundles + vascular tissue (PSVB), mesophyll (MES) and total epidermis (TEpi)) and nutritive value (crude protein (CP), in vitro dry matter digestibility (IVDMD) and ash (ASH)) characteristics of leaves with contrasting levels of insertion on the tiller axis (phytomers) of Napier elephant grass. Points represent the phytomers with their respective segments and arrows represent the vector for each variable.

Three principal components (PC) were selected according to the eigenvalues of the correlation matrix using the Kaiser criterion, which selects eigenvalues bigger than 1. The first principal component (PC1) explained 51.08% of the overall variation of the data set, the second (PC2) explained 23.55% and the third (PC3) explained 13.02%, totalling 87.65%. The arrangement of vectors on the biplot PC1 × PC2, which accounted for 74,63% of the overall variation in the data set, showed that longer leaf blades were characterised as follows: (1) Basal portions (quadrant 4)—great percentages of sclerenchyma (SCL (All anatomical tissue measurements were expressed as % of the cross sectional area of the leaf blade in each of the five portions of the leaves.)), central rib mass (CRM), central rib width (CRW) and mesophyll (MES) and small CP percentage, specific leaf area (SLA), IVDMD and total epidermis percentage (TEpi); (2) Middle portions (quadrant 1)—great values of parenchymatic sheath of vascular bundles + vascular tissue (PSVB), blade length (BL), segment length (SL), blade mass (BM), total mass (TM), blade width (BW) and total width (TW) and small values of ashes (ASH); (3) Apical portions (quadrant 2)—great values of CP, SLA, IVDMD and TEpi and small values of SCL, CRM, CRW and MES. In quadrant 3, grouping represented small leaves, regardless of portions or segments, which were characterised by great values of ASH and small values of PSVB, BL, SL, BM, TM, BW and TW (Fig. 5). On the biplot PC2 × PC3, which represented 64.10% of the overall variation in the data set, shorter leaves, despite being older, were characterised by great values of IVDMD, ASH, CP, TEpi and SLA (quadrant 2) and small values of PSVB, SCL, MES, BM, BL and SL (quadrant 4) (Fig. 6).

### Cluster analysis

**Figure 7:**
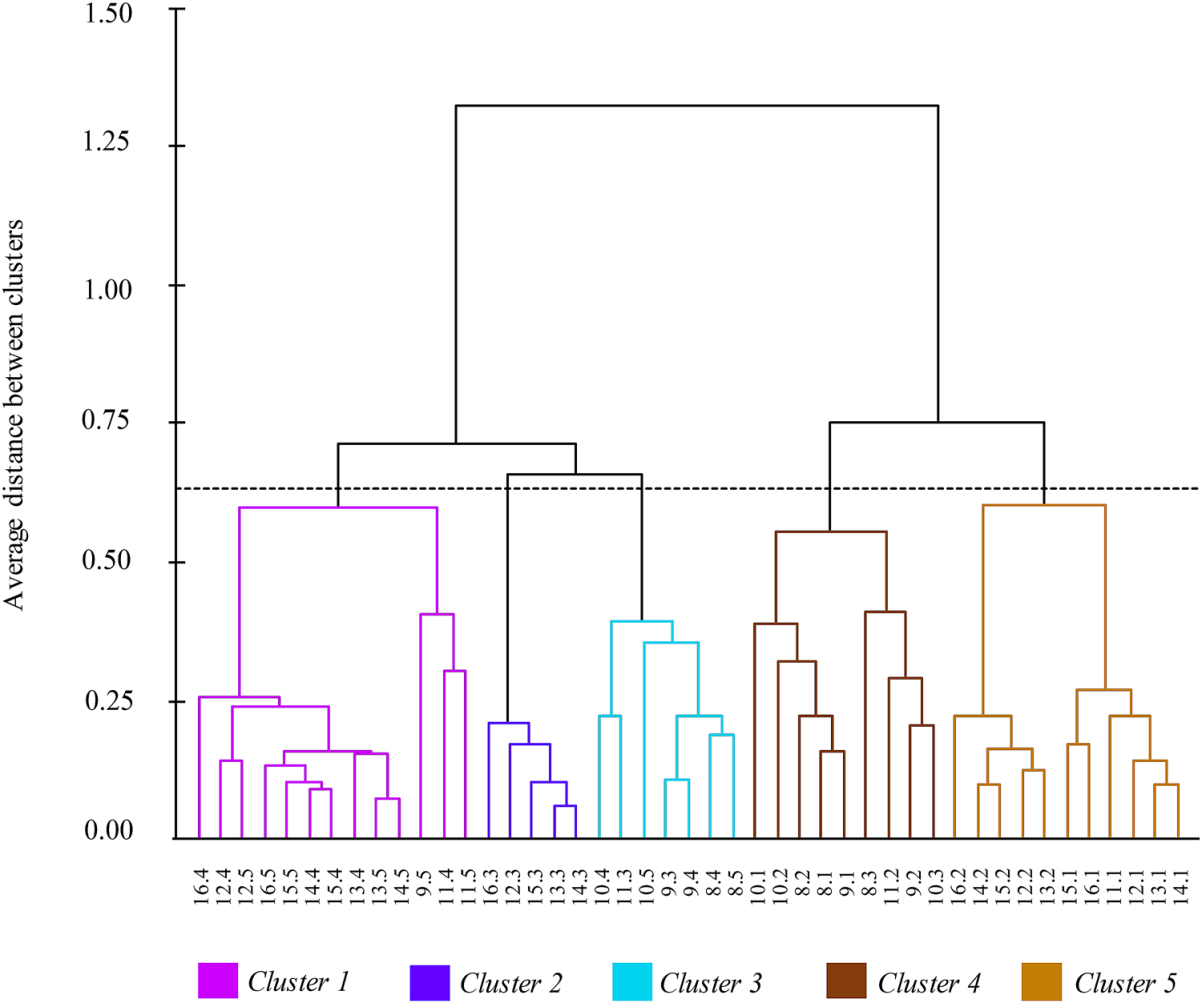
Dendrogram illustrating the groups formed by the cluster analysis using blade segments of leaves with contrasting levels of insertion on the tiller axis (phytomers) of Napier elephant grass.

Grouping from the Cluster analysis produced results in line with those of the PCA, which helped to their interpretation and discussion. Observing the dendrogram from left to right and using a reference cut of 0.60, it is possible to identify five well defined clusters based on the sum of the squares using the unweighted pair group method with arithmetic mean (UPGMA), according to which clusters are linked by the similarity among their elements. Clusters 1 and 3 comprised small phytomers (quadrant 3—biplot PC1 × PC2); cluster 2 comprised basal blade segments of phytomers 11 to 16 (quadrant 4—PC1 × PC2); cluster 4 comprised middle segments (quadrant 1—PC1 × PC2) and cluster 5 comprised apical segments of longer phytomers (quadrant 2—PC1 × PC2) (Fig. 7).

## Discussion

Napier elephant grass is tropical forage grass with a very high dry matter production potential commonly used in intensive pastoral dairy systems^24^. Understanding the relationship between morphological, anatomical and nutritive value traits is therefore strategic for planning grazing management strategies that ensure high quality herbage.

The linear relationship between total central rib mass and total blade mass (Fig. 2) emphasizes the coordination in allocation of mass for the central rib and the leaf blade (Table 1). Longer leaves were positioned at higher levels of insertion on tiller axis, since there was no internode elongation (apical meristem elevation) and greater total blade and central rib mass were recorded. The increase in central rib mass associated with the increase in total blade length according to leaf insertion level on the tiller (Table 1), particularly for the basal portion of the blade (Fig. 1), may have been consequence of greater need to structural support to maintain the erect architecture of leaves with an appropriate angle to intercept the incident light^12^, since that the central rib is the main leaf rib and, for some tropical grasses, it usually provides additional structural support^16^. Wheller et al.^17^, studying six varieties of commonly cultivated forage sorghum, reported that the central rib represented 21 to 28% of the total leaf blade weight, smaller values relative to those recorded in this study (30 to 41%), most likely because of morphological and bulk differences between forage sorghum and Napier elephant grass. Another possibility is that physiologically, longer leaves have longer hydraulic path lengths, and because path length influences the water potentially required to continually conduct water, both stems and leaves have evolved tapering of major vein xylem from the base to the tip of the leaf (from the base to the tip of the stem as well), which has evolved to overcome this path length constraint^18^.

Regarding the anatomical composition of the leaves, the literature describes that large leaves, with higher insertion levels on the tiller, show greater percentage of structural support tissues and cells with thickened secondary walls than small leaves, with lower insertion levels on the tiller^8, 25^. Wilson^25^, in a study with *Panicum maximum* var. trichoglume, reported a variation gradient on leaf tissue proportions along the tiller axis. Paciullo et al.^8^ also reported the same pattern of variation for *Brachiaria decumbens*, *Melinis minutiflora* and *Cynodon sp.* (Tifton-85).

The variation in leaf blade anatomy according to the insertion level on the tiller axis is a specific characteristic related to tiller development^25^ that may be observed in most grasses. The unique feature of this study is that it focused on studying the anatomical variations along the entire length of each leaf on the tiller axis (contrasting hierarchical order and size) and not only on the middle portion of the blade as usually made in protocols of this nature for comparing leaves with different levels of insertion on the tiller (e.g. age). There was a small variation gradient for mesophyll percentage from the base to the tip of the blade, with an average variation of 8% and greatest values recorded on the thickest segments of the blade (portions with smallest specific leaf area—SLA), where the percentage of central rib mass was greatest (Fig. 3a). Greatest percentage of sclerenchyma and vascular tissue were also recorded on the segments close to the base of the blades, ensuring stronger structural support (segments 1 and 2, Fig. 3b). Sclerenchyma and xylem cells are important for ensuring the erect architecture of leaves, particularly in warm and dry environments^13, 14^. Greater percentages of parenchymatic sheath of the vascular bundles and vascular tissue were recorded on the middle portion of the blades (Fig. 3c), possibly because of the greater blade width and smaller percentage of central rib mass relative to the basal portions of the blade. Greater percentages of epidermis (adaxial and abaxial) were observed in the apical portion of the blades (segments 4 and 5, Fig. 3d,e). The epidermis is comprised of live, vacuolated cells with the function of restricting water loss and, as a result, does not have intercellular space^26, 27^. In spite of the small anatomic composition variation along the length of the leaf blades, the distribution of anatomical tissues was relatively stable along the length of leaves of different sizes and levels of insertion on the tiller axis, i.e. the same pattern of variation in anatomical composition was observed for all phytomers regardless of their hierarchical order of development. These findings support the use of protocols based on measurements of the middle portion of the blades only, reducing the amount of field and lab work in studies of this nature.

When leaf age was taken into account (harvest of all leaves only when leaf 16 had completed expansion), the results were similar to those of leaves harvested individually soon after their full expansion (Fig. 4). This is probably because after complete expansion the anatomical composition of leaves is already defined and does not change with time, resulting in similar relative contribution of each anatomical tissue regardless of leaf age^25, 28, 29^. There are evidences in the literature consistent with our findings in which the effect of age and climatic factors on leaf anatomical composition were also small^19, 30^. Wilson^25^, in a study with *Panicum maximum* var. trichoglume, reported no variation in the proportion of anatomical tissues with increases in leaf age, but sclerenchyma cell wall was thicker, particularly in leaf sheaths positioned at the apical portion of the tillers (e.g. higher insertion levels). Similar results were reported for *Brachiaria decumbens*, *Melinis minutiflora* and *Cynodon sp.* (Tifton-85)^8^.

The central rib influenced IVDMD and CP along the length of the leaf blade, since when chemical analysis was performed on blade material without the central rib (e.g. taking into account only the anatomical composition of the blade), differences were too small and did not explain the differences in IVDMD and CP of whole leaves. Variability in IVDMD among C_4_ species is more dependent on the rate and extension of degradation of tissues like mesophyll, parenchymatic sheath of the vascular bundles and sclerenchyma than the amount of the different anatomical tissues^31^. As a result, the smaller IVDMD values for the basal segments (segments 1 and 2) of phytomers 12, 13, 14 and 15 and basal, mid-basal and middle segments (segments 1, 2 and 3) of phytomer 16 were probably consequence of the large percentage of central rib mass on those portions of the leaf blade (Table 4). The structural support assured by the central rib to the blade is mainly provided by the vascular tissue (xylem) and sclerenchyma^5^. These tissues are formed by cells from the thickened secondary wall and are the main responsible for the reduction in forage nutritive value^15, 16^, since they form a solid multicellular block in the rumen that results in large particles of difficult digestion because of lignification and associated restrictions to colonisation by rumen microbes^15, 32^. As the central rib usually contains large percentage of vascular tissues and sclerenchyma, its digestibility is significantly smaller than the blade^17, 33^. As a result, the basal segments of leaves of higher insertion levels on the tiller (e.g. bigger leaves), because of their larger mid rib mass, show smaller IVDMD than the middle and apical in spite of their greater percentage of mesophyll, one of the most easily digested plant tissues^34^.

Although leaves 8, 9, 10 and 11 showed smaller IVDMD on the apical portions, the average IVDMD for the whole leaf blade of those phytomers was 82.22; 83.05; 82.08 and 82.12%, respectively. For the phytomers of higher level of insertion on the tiller (phytomers 12, 13, 14, 15 and 16), the average IVDMD values of middle, mid-apical and apical segments (segments 3, 4 and 5, respectively) were 82.71; 82.24; 82.63; 82.68 and 82.82%, respectively, similar to those of phytomers of lower level of insertion despite these being older. The smaller IVDMD of the apical segments of leaves 8, 9, 10 and 11 may have been consequence of their advanced maturity, since the stage of development is the most important factor influencing the nutritive value of forage grasses^35^. The apical segment of the blades for phytomer 8 and part of the blades for phytomer 9 showed evident signs of senescence like yellowing, darkening and tissue degradation due to remobilization of nutrients to other growing points, a condition that is well known for reducing the nutritive value of leaves^35, 36^. On the other hand, phytomers 5, 6 and 7 were completely senesced at the time of harvest when leaf 16 completed expansion. If only the green portion of the blades is considered, with no export of nutrients to growing points (i.e. leaf senescence), it is possible to verify the negative impact of the central rib on the digestibility of leaf blades.

The greatest CP value was recorded on the apical segment (segment 5) of phytomer 16 (Table 5), and did not differ from those segments with smaller percentages of central rib mass and greater values of SLA. These findings are in line with those of Lucena et al.^23^, which indicated positive correlations between CP and SLA (r = 0.52; *P* = 0.0010) on leaf blades of *Paspalum* spp.. The difference in CP detected on segment 5 of phytomer 10 may have been consequence of leaf maturity stage, similarly to all segments of phytomers 8 and 9, since cell wall components and fibre increase and digestibility and crude protein decrease as leaf age increases^7, 14^. Protein, along with energy, is the most required nutrient by ruminants, and the smaller CP content observed in this study (11%) is significantly greater than the minimum recommended of 7% for adequate ruminal fermentation^37^. Similar to the IVDMD results, central rib exerted a negative effect on CP content, possibly because of the reduction in blade proportion, where tissues with greater nitrogen content like mesophyll and parenchymatic sheath of the vascular bundles are present.

The findings of the multivariate analysis (Cluster and PCA) corroborated and integrated the results from single variable analysis (ANOVA), since they generated clusters and principal components according to the level of insertion of phytomers on the tiller axis and their structural support (e.g. blade length associated with central rib distribution along the blade), relating morphological, anatomical and nutritive value characteristics. On the PCA-derived biplots, quadrants are inversely correlated (vectors in opposite directions). Large vectors indicate large variability in the data set, and proximity among vectors indicates how strongly related they are (Figs. 5, 6). The basal portions of blades from phytomers of high level of insertion on the tiller axis were characterised by structural support variables (SCL; CRM and CRW), in line with the morphological data (greater central rib mass). Additionally, they were associated with greater mesophyll percentage, probably because they were thicker leaves (smaller SLA) and, consequently, of lower nutritive value (Fig. 5, quadrant 4). According to the literature, SLA is usually related to leaf anatomy, with large SLA associated with thinner cuticles, epidermis and mesophyll^38, 39^. The middle portions of the blades corresponded to the regions where blade width was greater, consequently with greater concentration of vascular bundles resulting in greater blade mass relative to the entire length of the leaf (Fig. 5, quadrant 1). The apical portions were thinner, with larger SLA and higher nutritive value, possibly because of their smaller central rib mass (Fig. 5, quadrant 3).

In general, phytomers of lower insertion level on the tiller (smaller leaves), although older, were characterised by nutritive value variables (IVDMD, CP and ASH), TEpi and SLA. In spite of the slow and partial digestion of the epidermis from tropical grasses^15^, this variable was associated with regions of higher nutritive value, in line with the findings of Wilson et al.^31^, according to which epidermis cells were rapidly and extensively degraded for the majority of the *Panicum* species studied. However, significant correlations between digestibility and epidermis percentage were not found^31^. The bulk of the characteristics associated with the smaller phytomers indicate the strong negative impact of the central rib on the nutritive value, particularly over the IVDMD, since the anatomical composition of the blades did not change with leaf age^5^.

## Conclusions

Leaf blades of Napier elephant grass show negative relationship between central rib and nutritive value, highlighting the importance of the central rib in determining the nutritive value of the herbage. The apical portions of the blades have similar digestibility to small leaves regardless of leaf chronological and physiological age, indicating that grazing management that favours the removal of the apical portion of leaves (moderate/lenient defoliation regimes) ensures the harvest of high nutritive value herbage by the grazing animals.

## Methods

All procedures were approved by the Animal and Environment Ethics Committees of the University of São Paulo, College of Agriculture “Luiz de Queiroz” (USP/ESALQ). All applicable international, national, and/or institutional guidelines for the care and use of animals were followed.

### Experimental site

Two experiments were carried out at Luiz de Queiroz College of Agriculture, University of São Paulo, Piracicaba, SP, Brazil (22° 42′ S, 47° 38′ W and 546 a.s.l.), during the summer 2017 (January to March). Napier elephant grass (*Pennisetum purpureum* Schum. cv. Napier) was used as model plant because of its large size, ease of vegetative propagation and the nature of the study. The soil was a high fertility Eutric Kandiudalf with the following chemical characteristics for the 0–20 cm layer: **Experiment 1**—pH CaCl_2_ = 5.9; OM = 46.0 g dm^−3^; *P* (ion-exchange resin extraction method) = 257.0 mg dm^−3^; Ca = 148.1 mmolc dm^−3^, Mg = 80.0 mmolc dm^−3^; K = 9.1 mmolc dm^−3^; H + Al = 15.0 mmolc dm^−3^; sum of bases = 237.1 mmolc dm^−3^; cation exchange capacity = 252.1 mmolc dm^−3^; base saturation = 94%; **Experiment 2**—pH CaCl_2_ = 5.8; OM = 39.3 g dm^−3^; *P* (ion-exchange resin extraction method) = 54.0 mg dm^−3^; Ca = 62.0 mmolc dm^−3^, Mg = 22.3 mmolc dm^−3^; K = 8.6 mmolc dm^−3^; H + Al = 29.0 mmolc dm^−3^; sum of bases = 93.1 mmolc dm^−3^; cation exchange capacity = 122.0 mmolc dm^−3^; base saturation = 76%. These were considered adequate for the forage species used, with no need for additional fertilisation.

The climate, according to Köppen classification, is Cfa, humid subtropical climate with wet summer^40^ and an average annual rainfall of 1,328 mm. The average air temperature during the experimental period was 24.2 °C and total precipitation 753.85 mm, from which 424.4 mm corresponded to total precipitation for Experiment 1 (Dec 27, 2016 to Feb 21, 2017) and 502.14.5 mm for Experiment 2 (Dec 09, 2016 to Mar 14, 2017). The greatest precipitation was recorded in January 2017 (336.55 mm).

To avoid soil water deficits, a drip irrigation system was installed in the area used for Experiment 1 and a sprinkler irrigation system was available in the area for Experiment 2. Irrigation in both areas was carried out according to records of precipitation, average air temperature and evapotranspiration. On rainy days, precipitation was recorded and taken into account in calculations for irrigation as a means of ensuring that plants were not submitted to either deficit or excessive soil moisture.

### Experiment 1 (leaf morphology and anatomy): establishment and experimental control

Preparation of the experimental area (290 m^2^) started with the desiccation of previous vegetation (*Cynodon dactylon* (L.) Pers.) using the broad-spectrum herbicides Glyphosate (N-phosphonomethyl-glycine) and 2.4-D (2.4-Dichlorophenoxyacetic acid) in Sept 09 and Nov 15, 2016, and Paraquat (1.1′-dimethyl-4.4′-bipyridinium dichloride) in Dec 11, 16 days before planting on Dec 27, 2016.

One day before planting of the experimental area (290 m^2^), planting pits were opened and the desiccated vegetation around them removed. Planting material (stem cuttings with viable lateral buds) was harvested at an 850 m^2^ pasture of well-established Napier elephant grass^41^. Stems were fractioned in one-node pieces (one single axillary bud) discarding the basal and the apical portions of the stems to ensure vigorous sprouting from the planting material. Ten buds were placed in each pit at 5 cm depth, covered with soil and gently compressed by hand. The distance between pits in lines was 1.5 m and between planting lines was 2 m, in order to obtain the desired spaced-plant layout. Two weeks after planting, plants were thinned leaving one plant per pit.

Treatments corresponded to phytomer order along the tiller axis and the experimental design was a randomised complete block, with four replications. Plants within blocks were randomised using the statistical package SAS (Statistical Analysis System, v. 9.0).

Weed control during the experiment was carried out manually. Pest (*Mocis* sp.) and disease (*Bipolaris* sp.) control was carried out using the water-soluble insecticide Resolva (Lambda-Cyhalothrin) in Jan 07 and 22, 2017 (5 g L^−1^) and the fungicide Nativo (Trifloxistrobina + Tebuconazol) in Feb 05, 2017 (0.6 L ha^−1^), respectively.

Sampling followed the ontogenetic programme of plants, beginning with tiller 1, which corresponded to the anatomical evaluation of the 8th leaf (phytomer 8); tiller 2, which corresponded to the anatomical evaluation of the 9th leaf (phytomer 9) and the morphological evaluation of the 8th leaf; tiller 3, which corresponded to the anatomical evaluation of the 10th leaf (phytomer 10) and the morphological evaluation of the 9th leaf in this sequence until full expansion of the 16th leaf (phytomer 16) for morphological evaluation (tiller 10), totalling 40 tillers (4 tillers for anatomical characterisation of the 8th expanded leaf + 32 tillers for anatomical (9th to 16th leaf) and morphological (8th to 15th expanded leaf) evaluations + 4 tillers for morphological characterisation of the 16th expanded leaf). At each harvest, leaves were carefully removed from the tillers, identified and preserved with ice until processing in the laboratory. Sampled tillers were removed from the experimental area. In order to evaluate the effect of leaf age on the deposition of support tissues, all leaves from 8 tillers (4 for anatomical evaluations (tiller 11) and 4 for morphological evaluations (tiller 12)) were collected at a single harvest when the 16th leaf completed expansion following the same procedure described for each leaf separately. Twenty additional plants were grown (five per block) to ensure that all leaves would be harvested as planned, but they were not necessary.

#### Leaf anatomy

In the laboratory, the leaf blade was cut at the ligule, its length was measured (distance between the tip of the leaf and the ligule) and fractionated in five segments of similar length designated as: (1) basal—closest portion to the insertion on the tiller; (2) mid-basal—middle portion closest to the basal; (3) middle—middle portion of the blade; (4) mid-apical—middle portion closest to the apical; (5) apical—portion closest to the tip of the leaf. Each of the five segments were fragmented in 1-cm cuts and stored according to methodology described by Johansen^42^. Sample dehydration was carried out using a progressive alcoholic series with tertiary butyl alcohol^43^, and fragments infiltrated with paraffin and subsequently with paraplast. In sequence, fragments were sectioned (12-µm width) with a Leica Biosystems manual rotary microtome, followed by a triarch quadruple staining of tissues before permanent blade mounting, following the methodology proposed by Hagquist^44^. Images were captured using the AxioVision Program (V2.05, Carl Zeiss Vision) attached to a Zeiss Axioskop 2 binocular optical microscope and a Zeiss AxioCam MRc (1.388 × 1.040 pixels) digital camera. Images were captured using 20× objective lens from the first large vascular bundle after the central rib as a means of standardising readings. Estimates of the percentage of each anatomical tissue on the samples were made using the AxioVision software (AxioVs40, release 4.8.2.0, Carl Zeiss Micro Imaging GmbH, Germany). Initially, the whole cross-section area projected on the video was measured (STotal). Next in the measurement sequence were the areas of adaxial (EPIada) and abaxial (EPIaba) epidermis, parenchymatic sheath of vascular bundles (PSV), vascular tissue (VT—including xylem, phloem, mestome sheath and pericyclic fibers^27^) and sclerenchyma (SCL). The mesophyll area was calculated as the difference between STotal and that of all the other tissues, therefore including airspace. Measurements were made in µm^2^ and the results expressed as percentage of total area.

#### Leaf morphology

For the morphology measurements the leaf was cut at the ligule, its length was measured (distance between the tip of the leaf and the ligule) and fractionated in ten segments of similar length. At the mid portion of each segment the fragment width (distance between the opposite borders) was measured in millimetres. In the sequence, the central rib from each segment was removed using a scalpel and its width and length were also measured. The fragment parts (central rib and blade tissue) were passed through a LAI-3100 leaf area integrating device (LI-COR) and put to dry in a forced draught oven at 55 °C until constant weight. The results were used to calculated whole segment mass (mg) and specific leaf area (SLA—cm^2^.mg^−1^).

### Experiment 2 (nutritive value): establishment and experimental control

**Figure 8:**
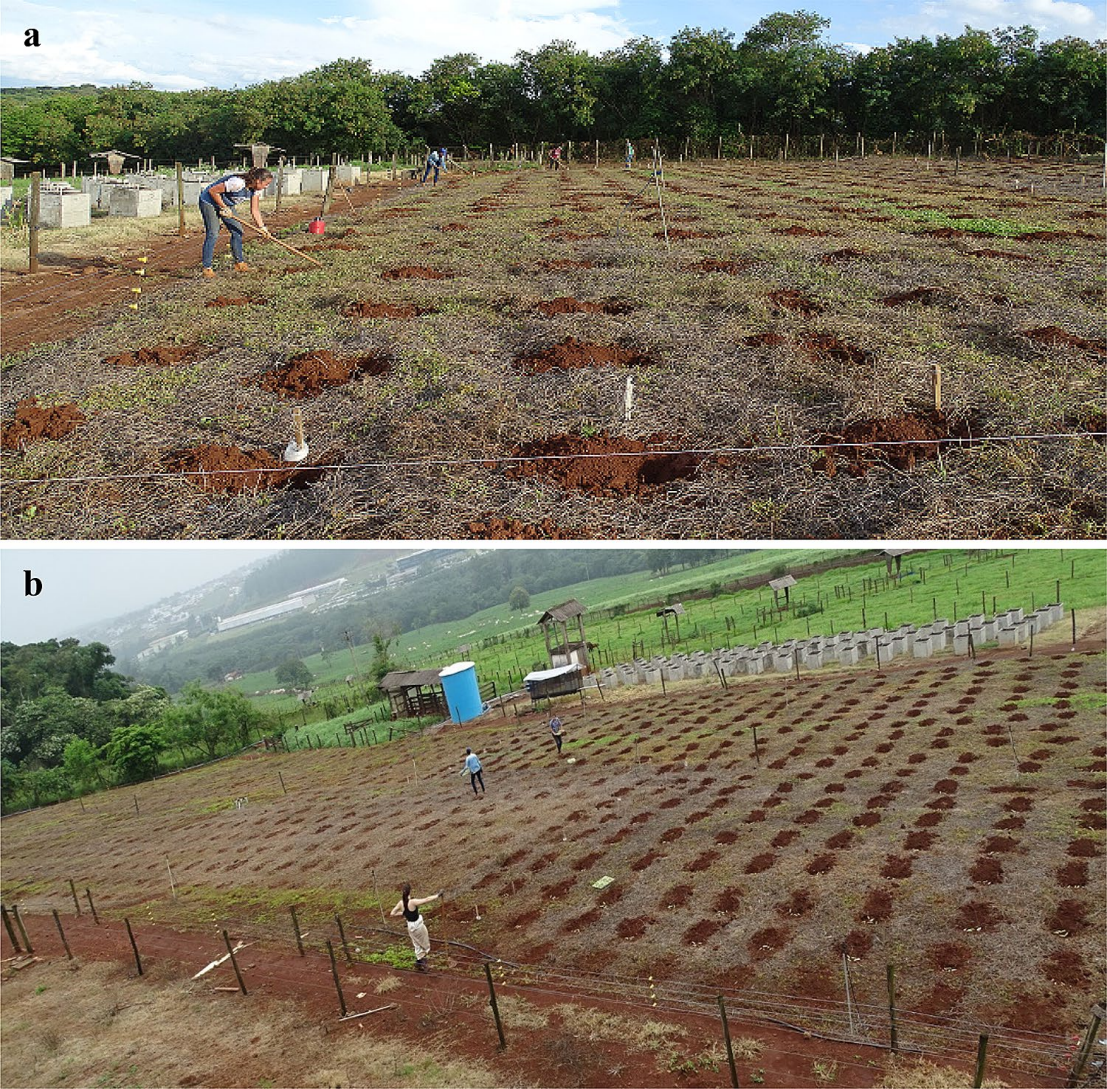
General view of the experimental site during the establishment phase showing the layout and distribution of elephant grass plants on the area: (**a**) Pit opening and (**b**) planting of the stem cuttings.

Preparation of the experimental area (3,000 m^2^) started with the desiccation of previous vegetation (*Arachis pintoi* cv. Belmonte) using the broad-spectrum herbicides Glyphosate (N-phosphonomethyl-glycine) and 2.4-D (2.4-Dichlorophenoxyacetic acid) in Sept 08, Oct 01 and Nov 15, 2016. On Nov 30 the whole area was mowed and a final application of Paraquat (1.1′-dimethyl-4.4′-bipyridinium dichloride) was carried out in Dec 06 (blocks 2 and 3) and Dec 11, 2016 (block 1). The next steps followed the same protocol used in Experiment 1. Six buds were placed in each pit at 5 cm depth, covered with soil and gently compressed by hand. The distance between pits in lines and between lines was 1.0 m (Fig. 8).

A total of 1,320 plants were cultivated. These were divided in three homogeneous blocks with 440 plants each. The number of plants per block was dimensioned to provide the necessary amount of dried and ground samples for the nutritive value analysis. Twenty days after planting, plants were thinned leaving one plant per pit.

Treatments corresponded to phytomer order along the tiller axis and the experimental design was a randomised complete block, with three replications. Plants within blocks were randomised using the statistical package SAS (Statistical Analysis System, v. 9.0). Weed, pest and disease control was the same as for Experiment 1.

Sampling was carried out when the 16th leaf (phytomer 16) complete its expansion and exposed the ligule. Main tillers had all their leaves identified with permanent marker before harvest. The leaves were stored in ice and taken to the laboratory. Processing involved removal of the sheaths and blades stored in freezer for future segmentation. Leaves 5, 6 and 7 were discarded because they were in advanced stage of senescence and decay, leaving leaves 8 to 16 for the analysis.

#### Nutritive value

After all field work was finished, leaf blades had their length measured (distance between the tip of the leaf and the ligule) and were fractionated in five segments of similar length, as described for leaf anatomy measurements in Experiment 1 (i.e. chemical analyses included all tissues of the leaf blade). These were pooled into a composite sample per leaf hierarchical order, totalling 135 samples (9 leaves × 5 segments × 3 blocks). These were put to dry into forced draught oven at 55 °C until constant weight.

After drying, because the apical portion of the leaves was too delicate, the dried material was ground in a micro mill with a 1 mm sieve (Wiley Mill, Thomas Scientific, Philadelphia, PA, USA) as a means of reducing dry matter losses. Ground samples were subjected to the following chemical analysis: *in vtiro* dry matter digestibility (IVDMD), using the artificial rumen fermentation device DAISYII from ANKOM Technology Corporation^45^; total nitrogen (N_total_), determined by the Dumas combustion method using the Leco FP 528 System (Leco Instruments Inc., St. Joseph, MI, USA); and ashes (ASH) determined according to Silva & Queiroz^46^. Crude protein (CP) was calculated as N_total_ × 6.25. For the in vitro trial, rumen liquid was collected from one rumen-cannulated Nellore steer fed only with Tifton-85 (*Cynodon dactylon* spp.) haylage.

### Statistical analysis

Leaf blade morphology and anatomy data were initially analysed using descriptive statistical analysis (means and standard error of the mean). Leaf blade total mass and central rib total mass were obtained by adding values for all ten segments from each leaf and were subjected to a regression analysis as a means of identifying the correlation between these two variables. Regression was performed using the procedure PROC REG of SAS (Statistical Analysis System, v. 9.0).

Nutritive value data were subjected to ANOVA using the procedure PROC GLM of SAS (Statistical Analysis System, v. 9.0), and means compared by Tukey test (*P* < 0.05). The statistical model considered phytomer and block as fixed effects:

$$Y_{ij} = \mu + B_{i} + F_{j} + \varepsilon_{ij}$$

where Y_*ij*_ : average response of the *j*th phytomer in the *i*th block; µ : mean; B_*i*_ : block *i*; F_*j*_ : phytomer *j*; ε_*ij*_ : random error ~ NID (0;σ^2^_ε_). All assumptions for these analyses were verified as homogeneity of variances, error normality, outliers, and need for transformation by Box–Cox^47^.

A Principal Components Analysis (PCA) was carried out with the objective of integrating the leaf morphology, leaf anatomy and nutritive value results. The data set was comprised of the following response-variables: Morphology—blade length, segment length, blade width, central rib width, total width (blade + central rib), blade mass, central rib mass, total mass (blade + central rib) and specific leaf area; Anatomy (percentage of the cross sectional areas for each tissue)—mesophyll, vascular bundles (parenchymatic sheath of vascular bundles + vascular tissue), total epidermis and sclerenchyma; and Nutritive Value—crude protein, in vitro dry matter digestibility and ash (data from Experiment 2 when all leaves were harvested at full expansion of leaf 16). Because leaf blades were fractioned in ten segments for morphology measurements and five segments for anatomy and nutritive value measurements, segments from the morphology measurements were combined two-by-two to balance segment number from all measurements (1 + 2, 3 + 4, 5 + 6, 7 + 8 and 9 + 10 to form 1, 2, 3, 4 and 5, respectively). As a result, all response variables had blade segments associated with them. PCA was performed using the procedure PROC PRINCOMP and the biplots were generated using the procedure PROC PRINQUAL of SAS (Statistical Analysis System, v. 9.0).

Cluster analysis was performed using the same PCA data set with the unweighted pair-group method with arithmetic mean (UPGMA) using the procedure PROC CLUSTER of SAS (Statistical Analysis System, v. 9.0) as a means of identifying phytomers groups according to the similarity of their elements.

## Data Availability

The datasets generated during and/or analysed during the current study may be available from the corresponding author upon request.
